# Traffic Light Recognition Based on Binary Semantic Segmentation Network

**DOI:** 10.3390/s19071700

**Published:** 2019-04-10

**Authors:** Hyun-Koo Kim, Kook-Yeol Yoo, Ju H. Park, Ho-Youl Jung

**Affiliations:** 1 Department of Information and Communication Engineering, Yeungnam University, Gyeongsan 38544, Korea; kim-hk@ynu.ac.kr (H.-K.K.); kyoo@ynu.ac.kr (K.-Y.Y.); 2 Department of Electrical Engineering, Yeungnam University, Gyeongsan 38544, Korea; jessie@ynu.ac.kr

**Keywords:** advanced driver assistance system, artificial neural networks, binary semantic segmentation, deep learning, traffic light detection, traffic light recognition

## Abstract

A traffic light recognition system is a very important building block in an advanced driving assistance system and an autonomous vehicle system. In this paper, we propose a two-staged deep-learning-based traffic light recognition method that consists of a pixel-wise semantic segmentation technique and a novel fully convolutional network. For candidate detection, we employ a binary-semantic segmentation network that is suitable for detecting small objects such as traffic lights. Connected components labeling with an eight-connected neighborhood is applied to obtain bounding boxes of candidate regions, instead of the computationally demanding region proposal and regression processes of conventional methods. A fully convolutional network including a convolution layer with three filters of (1 × 1) at the beginning is designed and implemented for traffic light classification, as traffic lights have only a set number of colors. The simulation results show that the proposed traffic light recognition method outperforms the conventional two-staged object detection method in terms of recognition performance, and remarkably reduces the computational complexity and hardware requirements. This framework can be a useful network design guideline for the detection and recognition of small objects, including traffic lights.

## 1. Introduction

A traffic light (TL) recognition system is a very important building block in an advanced driving assistance system (ADAS) and an autonomous vehicle system [1,2,3]. Information from various sensors, such as a stereo camera, radar sensor, digital-map, and GPS, are combined to improve recognition performance by predicting the position of the TL [4,5,6,7,8,9,10,11,12]. Though the sensor fusion methods give better performance, they suffer from high sensor costs, high computational complexity, and sophisticated manipulations such as sensor calibration and spatio-temporal synchronization among the various sensors [4,5,6,7,8,9,10,11,12]. For these reasons, research to improve the recognition performance using a single RGB camera has been continuously and thoroughly conducted in the literature [13,14,15,16,17,18,19,20,21,22,23,24,25,26,27]. Many methods have been developed based on signal processing and computer vision techniques, but they are still highly sensitive to environmental variations such as illumination change and noise.

Recently, deep-learning methods, such as region-convolutional neural network (R-CNN) [28], Fast R-CNN [29], Faster R-CNN [30], region-based fully convolutional networks (R-FCN) [31], Mask R-CNN [32], you only look once (YOLO) [33,34,35], and single shot multibox detector (SSD) [36,37] have showed innovative performance in the object recognition field. The first five methods are composed of two stages: region detection and object classification [28,29,30,31,32]. The other methods combine both tasks into a single stage [33,34,35,36,37]. The two-staged methods give better performance, with the sacrifice of increased computational complexity. On the other hand, the single staged methods can detect objects in real-time, but produce low detection performance.

The above-mentioned methods cannot produce sufficient performance for detecting small TLs for an ADAS application, because they are originally developed for detecting general objects such as vehicles, pedestrians, and animals [38,39,40]. For the well-known Bosch traffic dataset [26], 89% of TLs are classified as small objects [40]. It should be noted that an object with less than 32 × 32 pixels is defined as a small object in the COCO dataset [41].

The deep-learning approach has deep depths of multiple layers with convolutional filtering and follows pooling, i.e., down-sampling. The deep layers produce various excellent features by using a large receptive field, i.e., the whole input image. For the TL recognition case, the large receptive field should be adjusted, because the TL has very small spatial resolution. Considering the intrinsic performance limitation of the single-staged approaches, it would be important to reduce the computational complexity of the two-staged approaches for a TL detection application.

In this paper, we propose a two-staged deep-learning-based traffic light recognition method that consists of a pixel-wise semantic segmentation technique and a novel fully convolutional network (FCN). The proposed method works in a manageable computational complexity and with sufficient recognition performance. For the detection of small objects, pixel-wise semantic segmentation [42,43,44,45] is applied to detect the TL candidate regions. To remove the computationally-demanding candidate detection and regression operations in the conventional two-staged approach, a region segmentation method in computer vision is adopted for real-time processing. By contrast, in the case of classification of TL types, we notice two important facts: (1) the resolutions of candidate TL regions are variable, unlike that of conventional deep-learning-based classification with a fixed input resolution, and (2) the TLs have only a set number of colors, such as red, green, yellow, and black (TL back-plate). The pre-processing of R-CNN [28] for input resolution variation is adopted in the proposed classification, i.e., the input region is warped to have the required resolution. For the proper color space transformation, (1 × 1) convolution layers are applied at the first layer in the TL classifier. The remaining network in the classifier is designed with a FCN considering the computational complexity and accuracy performance. The well-known Bosch traffic dataset is used for training and performance evaluation of the proposed method. The performance of the proposed method is compared with conventional two-staged TL recognition methods in terms of TL candidate detection and recognition performances, hardware requirements, and computational complexity.

The rest of this paper is organized as follows. In Section 2, we describe the proposed TL recognition method. In Section 3, the proposed method is empirically analyzed for various performance metrics, and the performance of the proposed method is compared with the conventional method. Section 4 draws the conclusions.

## 2. Proposed Traffic Light Recognition Method

**Figure 1:**
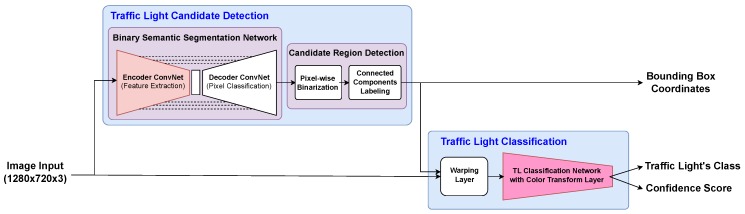
Proposed two-staged traffic light recognition method.

In this section, we present a two-staged deep-learning based TL recognition method that consists of candidate detection and classification stages, as shown in Figure 1. TL candidate regions and their positions are extracted in the candidate detection stage. For the classification stage, the candidate regions are discriminated into types of TLs, including background. The following subsections describe the two stages in detail. In addition, the training and inference processes are also described.

### 2.1. Traffic Light Candidate Detection Stage

Conventional object detection (OD) methods are not suitable to detect small objects, because they use very deep depth networks with pooling operations for feature extraction [46,47,48,49,50]. For example, Faster-RCNN employs anchor boxes to extract object candidate regions from a feature map. As the feature map is obtained by deep ConvNet, it has too wide a receptive field to reflect the existence of small objects. For the anchor box layer, additional memory is required according to the maximum number of object candidates. In addition, bounding box regression and non-maximum suppression (NMS) [51] are needed to calculate the precise location of a candidate region and to remove overlapping candidate regions, respectively [30]. These operations make it difficult to implement real-time processing.

The main idea of the proposed candidate detection is to employ a pixel-wise semantic segmentation that is applicable to very small objects. The proposed TL candidate detection stage consists of binary semantic segmentation and candidate region detection. Through the binary semantic segmentation, a confidence score is assigned to each pixel of an input image. The confidence score represents the possibility that each pixel belongs to the traffic light region. For the semantic segmentation, an FCN with an encoder-decoder structure can be used. In this work, we apply E-Net [45], which is efficient for both computational complexity and small object segmentation. Hereafter, the E-Net-based binary semantic segmentation is denoted as BSSNet.

In the detection of a candidate region, the bounding box of the region is calculated. The binary image is obtained by thresholding the confidence score of each corresponding pixel from BSSNet. To extract as many TL candidates as possible, all non-zero confidence scores are segmented as candidates. It should be noted that high threshold values may cause valid TLs to be excluded in the classification stage. Then, eight-connected-neighborhood-based connected components labeling (CCL) [52] is applied to the binary image to obtain separate candidate regions. The bounding box coordinates ($x_{min}\left(i\right)$, $y_{min}\left(i\right)$, $x_{max}\left(i\right)$, $y_{max}\left(i\right)$) of the *i*th candidate region are calculated, where [$x_{min}\left(i\right)$ and $y_{min}\left(i\right)$] and [$x_{max}\left(i\right)$ and $y_{max}\left(i\right)$] are coordinates of the top left and bottom right corners, respectively.

Unlike conventional two-staged ODs, the proposed candidate detection method does not require an anchor box layer and NMS operations. Therefore, the proposed method can be implemented with a relatively small memory and low computational complexity.

### 2.2. Traffic Light Classification Stage

**Figure 2:**
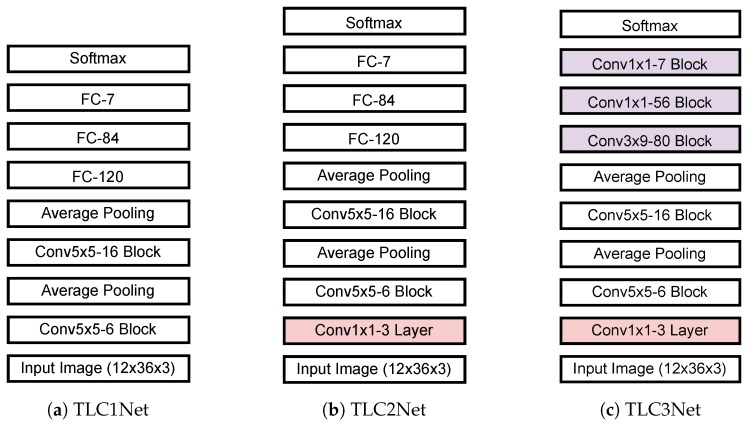
Proposed traffic light classification networks.

The TL classification stage classifies the types of traffic lights by using an input image corresponding to a candidate region obtained from the candidate detection stage. The stage consists of a warping layer and a TL classification network. It is observed in our evaluation dataset that most TLs are composed of three lamps. Considering the average width and height of traffic lights as shown in Table 1, the candidate region is cropped from the input image and wrapped to the size of 12 × 36 pixels. 89.01% of TLs belong to small size with less than $32^{2}$. The TL classification network classifies TL candidates into seven types such as red, red-left, green, green-left, yellow, off, and background. Considering the input image size and the number of classes, an LeNet-5-based [53] TL classification network is designed. Three TL classification networks are proposed and implemented, as shown in Figure 2.

The first network, hereafter referred to as TLC1Net, consists of two convolution blocks and three fully connected (FC) layers, as shown in Figure 2a. Each convolution block is composed of a convolution layer, a batch-normalization layer, and an activation function, in consecutive order. In each convolution block, a convolution layer with K filters of (N × M) is applied, which is denoted as the Conv N × M-K block. Unlike the LeNet-5 [53], the TLC1Net applies zero padding and ReLU [54]. In addition, batch normalization is applied between the convolution layer and the activation layer. Average pooling with factor 2 is applied after the first and second convolution blocks. The second proposed classification network, TLC2Net, is designed by adding a convolution layer with three filters of (1 × 1) to TLC1Net, as shown in Figure 2b. The additional layer is applied directly to the three-color channels of input data to perform an effective color space transform. As mentioned previously, a TL appears mainly with four colors such as red, green, yellow, and black. If we apply a color space that distinguishes the colors well, we can improve the classification performance. Although various existing color transforms can be applied before the classification network as in our previous works [40], effective color space transform coefficients are obtained through training processes. No bias weight is applied for the color transform layer. Figure 2c shows the third proposed TL classification network, TLC3Net, that is designed with FCN. As the three FC layers in TLC2Net are replaced by three convolution blocks, TLC3Net is faster than the TLC2Net, while reducing weight parameters. All three proposed TL classification networks have softmax [55] at the end to discriminate the type of TLs based on the confidence scores of the seven classes.

### 2.3. Training Process

A multi-task training process is carried out for TL candidate detection and classification. In the training task of TL candidate detection, the input data of the binary semantic segmentation network is an RGB color image, and the ground truth (GT) is a corresponding binary image in which only the pixels in the traffic light regions are “1”. As softmax is applied as the activation function at the last layer, cross-entropy loss [55] is used as the objective function. The network is trained until a maximum of 2000 epochs. During training, an adaptive moment estimation solver called Adam [56] is applied with a batch size of four, learning rate $10^{-4}$, and momentum parameters $\beta_{1}=0.9$, $\beta_{1}=0.999$, and epsilon(ϵ) = $10^{-8}$. Early stopping [57,58] is applied with patience parameter 50 of the validation loss minimum.

In the training task of TL classification, a previously-trained result in the binary semantic segmentation network is used. TL candidate regions extracted through the candidate region detection are cropped from the input image and wrapped to the size of 12 × 36 pixels. The resized TL candidate region is used as an input of the TL classification network. At this time, intersection over union (IoU) [59] is calculated by comparing the coordinates between the candidate region and the GT. If IoU is greater than or equal to 0.5 (IoU ≥ 0.5) [28,29,30,31,32,33,34,35,36,37,41,59], the TL candidate region is trained as the corresponding class of the GT. In contrast, if the IoU is less than 0.5 (IoU < 0.5) or there is no TL, the TL candidate region is trained as background. As the last layer of the TL classification network also uses softmax, cross-entropy loss is applied. The classification network is trained until the maximum of 200 epochs. During training, an Adam is also applied with learning rate $10^{-4}$ and momentum parameters $\beta_{1}=0.9$, $\beta_{1}=0.999$, and epsilon(ϵ) = $10^{-8}$. For TL classification training, the batch size varies depending on the number of TL candidates included in the given input image of driving road scenes. Early stopping is applied with patience parameter 10 of the validation loss minimum.

### 2.4. Inference Process

The TL candidate region is extracted from the RGB color image through the binary semantic segmentation network and the candidate region detection. The TL candidate region is resized into 12 × 36 size through the warping layer, and is classified into seven classes through the TL classification network. The class with the highest confidence score is finally selected. Except for the background class, the TL recognition outputs the type of TL, bounding box coordinates, and confidence score.

## 3. Simulation Environments and Performance Results

In this section, we evaluate the performance of the proposed TL recognition as compared with conventional two-staged deep-learning-based OD. For example, Faster R-CNN with inception-resnet-v2 [40] is compared. The performances are evaluated in terms of TL candidate detection and TL recognition. Before analyzing the performance, we briefly describe the dataset and measurement metrics for the performance evaluation in the following subsections.

### 3.1. Evaluation Dataset

For the simulations, we use an augmented version of the Bosch Small Traffic Lights Dataset [26] that is used in [40]. The evaluation dataset consists of 8144 RGB color images with 1280 × 720 resolution, and contains 17,102 annotated traffic lights. Six types of TLs are included, such as green, red, yellow, green-left, red-left, and off. The training and test datasets consist of 6102 and 2042 images, respectively, selected from 8144 color images. The proportion of training and test datasets of ’3:1’ are widely adopted throughout literature [60,61,62]. For performance comparison, we use the same data sets as in [40], which can be referred to [40] for more information. The test dataset is also used for validation.

### 3.2. Performance Measurement Metrics

TL candidate detection performance is evaluated by three metrics, such as precision, recall, and F-measure. For TL recognition performance, four metrics are used, such as average precision (AP), mean average precision (mAP), overall AP, and overall mAP. In addition, the average processing time is evaluated to verify the speed performance of the proposed TL recognition method. The network size according to the number of weight parameters is also compared.

### 3.3. TL Candidate Detection Performances

Table 2 shows the detection performances where the proposed BSSNet is applied for two different sizes of input image. In this table, BSSNet-full-size and BSSNet-half-size represent the BSSNet tested on input images of 1280 × 720 and 640 × 360, respectively. For these, BSSNet is trained independently for the two cases. Faster R-CNN with inception-resnet-v2 [40] is also tested for the input image of 1280 × 720 and compared. The performances are listed according to the sizes of the TL, i.e., small (# of pixel $\leq 32^{2}$) and non-small (# of pixels > $32^{2}$) [41]. A false negative indicates the cases in which a TL is not detected, or where the IoU is found to be less than 0.5 (IoU < 0.5). A false positive indicates the erroneous proposals where a background is misclassified as a TL candidate. The bold-marked numbers indicate the top-ranked method.

The proposed methods detect TL candidates better than Faster R-CNN in terms of all three metrics, i.e., precision, recall, and F-measure. BSSNet remarkably outperforms the conventional Faster-RCNN from the viewpoint of false positives. In particular, the proposed BSSNet has a relatively small number of false negatives. Note that a false negative has a direct effect on the performance of whole TL recognition system, as the region does not deliver to the TL classification stage. As expected, BSSNet-full-size has slightly better performance than BSSNet-half-size in terms of F-measure in total. This is caused by the fact that BSSNet-full-size detects small size TLs better than BSSNet-half-size, and the number of small size TLs is larger than the number of non-small ones.

### 3.4. TL Recognition Performances

The final performance of the proposed TL recognition is evaluated in terms of overall mAP and mAP@0.5, as shown in Table 3 and Table 4. As mentioned in Section 2.2, three TL classification networks such as TLC1Net, TLC2Net, and TLC3Net are applied and compared. BSSNet-full-size and BSSNet-half-size are applied to the three classification networks, respectively. A candidate region taken from a full-size input image is warped and fed to the classification network for both training and inference processes. In the case of BSSNet-half-size, the bounding box coordinates of the candidate region are scaled up by two, to crop the candidate region from the full-size input image. In the tables, the top-ranked method is marked with bold face.

In our previous work [40], three conventional methods, such as Faster R-CNN with inception-Resnet-v2 [63], Faster R-CNN with Resnet-101 [63], and R-FCN with Resnet-101 [63], are compared. Since the first method shows the best performance, it is used for the performance comparison in this paper and denoted as ’Faster R-CNN’. As compared to the Faster R-CNN method, the proposed TL recognition methods show significantly improved performances. In particular, the proposed TL recognition with BSSNet-full-size and TLC3Net improves performances by 24.1% in Overall mAP and by 31.68% in mAP@0.5, as compared with the conventional Faster R-CNN. It is observed that false positives are well-classified into background in the proposed TL classification networks. Among the proposed three classification networks, TLC3Net shows the best performance. TLC2Net produces improvements of 1.96% in overall mAP and 2.35% in mAP@0.5 over TLC1Net. It implies that the added convolution layer with (1 × 1) filters is particularly useful to extract the main color components of TL. TLC3Net produces improvements of 1.54% in overall mAP and 2.49% in mAP@0.5 over TLC2Net. It shows that the FCN is more useful than the FC layer for both complexity and performance. BSSNet-half-size-based TL recognition methods have also higher performance than Faster R-CNN at a much smaller computational complexity.

### 3.5. Performance Shift Analysis of TL Recognition

In this section, the performance variations are examined in terms of the ratio of training to test datasets, data swapping between the two datasets for a given ratio, and different database. For the first two evaluations, the proposed BSSNet-full-size and TLC3Net, which shows the best performance in Section 3.4, is used for the Bosch TL dataset. To know the performance variation by different database, the proposed BSSNet-full-size and TLC3Net is evaluated for the LISA Traffic Light Database [3,64]. The performance measures, mAP@0.5 is used for the analysis on performance variations.

**Figure 3:**
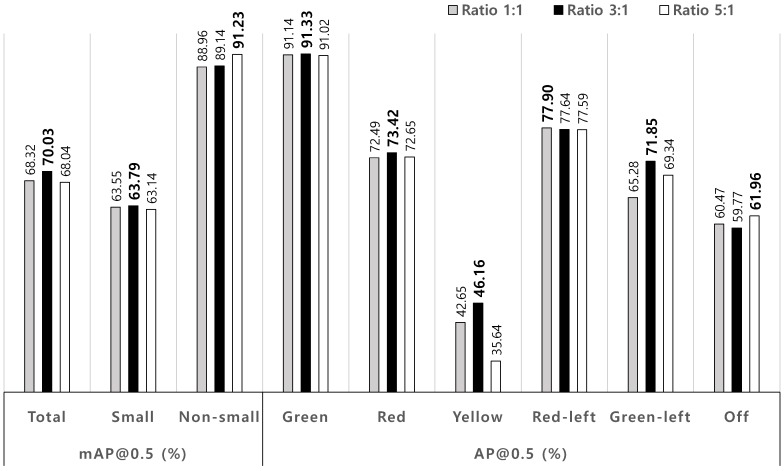
Average TL recognition performances according to ratio of training to test datasets.

For the evaluation of performance variations by ratios of training to test datasets, the selected ratios are 1:1, 3:1, and 5:1. Note that the total dataset is composed of 8144 images with 17,102 traffic lights from the Bosch TL dataset. In case of the ratio of 1:1, training and test datasets are composed of 4072 and 4072 images, respectively. For the ratios of 3:1 and 5:1, ’6102 and 2042’ and ’6783 and 1361’ images. For each simulation, 50% images in test dataset are randomly selected and swapped with training images. For each ratio of training to test dataset, the simulations are conducted by five times with the different swapped images. Average recognition performances are provided in Figure 3. The results implicate that the ratio of 3 to 1 gives the best performance, compared with other ratios but the performance variations are marginal. Performance shift by ratio dataset is only 1.99% in mAP@0.5, while the performance improvement of the proposed method over conventional method in mAP@0.5 is 31.55%. This implies that the performance shift is negligible with respect to the selection of swapped images. These tolerances to the variation of the ratios and image selections for training and test datasets come from the fact that the amount of data used in training is enough to train the small number of classes, i.e., the more than 4000 images for only six classes.

**Figure 4:**
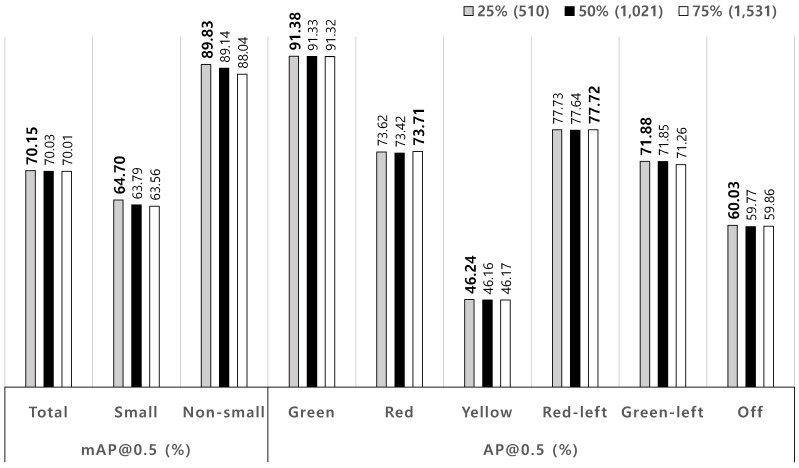
Average TL recognition performances according to dataset shift.

To evaluate the bias effect by selecting training data from the given total dataset, different proportions of test dataset are swapped with training dataset for a given ratio of training to test dataset, 3:1. For instance, the ’50% (1021)’ in Figure 4 means that 1021 images are swapped between training and test datasets.The swapping images are randomly selected for both datasets. For each proportion, the simulations are conducted by five times with the different swapped images. Figure 4 shows that the proposed method gives very robust performance to the selection of training and test datasets.

To evaluate validity to different database, the proposed and conventional methods are trained and tested on LISA Traffic Light Database [3,64]. The LISA database is obtained in the various environments during the daytime. It consists of 20,089 RGB color images with 55,536 annotated traffic lights. Six classes of TLs are go, go-left, warning, warning-left, stop, and stop-left. The database provides separate training and test datasets. The training and test datasets consist of 12,775 and 7314 images, respectively. Table 5 shows that the proposed method gives improvement in mAP@0.5 by 33.97%, compared with the conventional method. From the results on Bosch database in Section 3.4, the improvement in mAP@0.5 is 31.68%. The proposed method produces the similar amount of improvements for both databases. This result shows that the proposed method has the validity to different database.

### 3.6. Hardware Requirements

To analyze hardware requirements, we compare the network size according to the total number of weight parameters. We also analyze the size for each sub-network of the proposed TL recognition method. Table 6 shows the network size in megabytes (MB). The proposed TL recognition method requires approximately less than 1% of the network size of Faster-RCNN. In addition, the proposed recognition methods require slightly different network sizes depending on the TL classification network. The proposed BSSNet has the same number of weight parameters, even if the input size of the BSSNet is changed. Thus, BSSNet-x-size denotes both BSSNet-full-size and BSSNet-half-size in Table 6. The detailed network size for each sub-network in the proposed TL recognition method is summarized in Table 7. TLC2Net has very slightly larger network size than TLC1Net, as the color transform layer is added to the TLC1Net. TLC3Net has a relatively small size, because the FC layers are replaced by the FCN.

### 3.7. Computational Complexity

Computational complexity is evaluated by average processing time, as shown in Table 8. The inference processing time is measured on one Intel Core i7-6850K 3.60 GHz CPU and one NVIDIA Titan X Pascal GPU. Through simulations, it is observed that the proposed recognition methods have the same average processing time regardless of the TL classification network. This is caused by the fact that all three classification networks have almost the same number of weight parameters, as mentioned in Section 3.6. Then, the notation TLCxNet is used in Table 8.

The proposed TL recognition method with BSSNet-full-size is 5.47 times faster than Faster R-CNN. In the proposed methods, it takes the same average processing time for the candidate region detection (7 ms on CPU), warping operation (1 ms on CPU), and TL classification (1 ms on GPU), regardless of the input image size of BSSNet. It only takes different processing times for BSSNet-full-size (87 ms on GPU) and BSSNet-half-size (25 ms) because of the different sizes of the input image. As BSSNet is dominant factor for processing time, the proposed TL recognition method with BSSNet-half size can be implemented in real-time with the sacrifice of a minor decrease in recognition performance.

### 3.8. TL Recognition Examples

**Figure 5:**
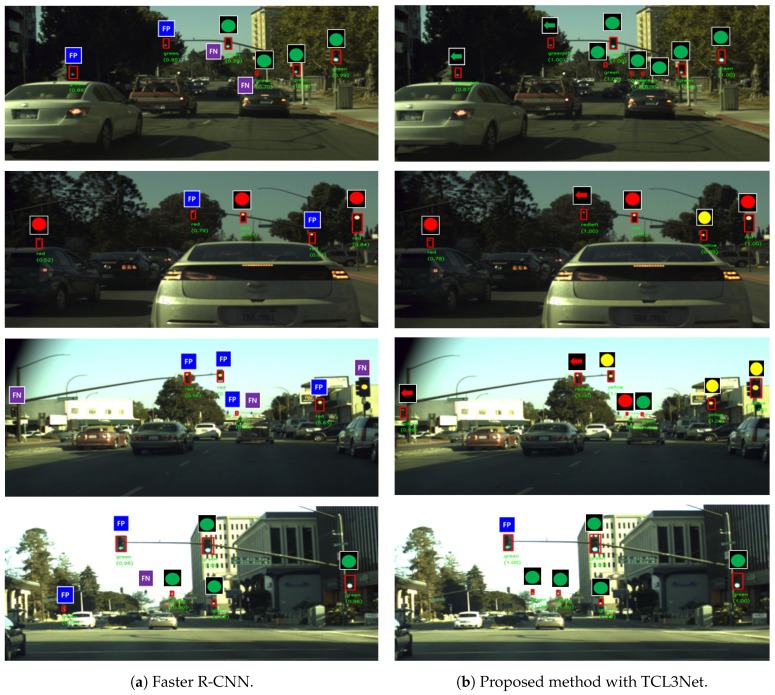
TL recognition examples of the Faster R-CNN and the proposed method.

Figure 5 shows the TL recognition examples of the proposed TL recognition method with the BSSNet-full-size and TLC3Net. Faster R-CNN examples are also shown. A true positive is indicated by the corresponding six types of TL symbol. False positive and false negative cases are indicated by a blue rectangle with FP and a purple rectangle with FN, respectively. As shown in Figure 5a, the Faster R-CNN has eleven true positives, nine false positives, and six false negatives in four images. Figure 5b shows that the proposed TL recognition method has twenty-five true positives and one false positive. As shown in the Figure 5, the proposed method has much better TL recognition performance than the conventional method. The first row in Figure 5 shows that the Faster R-CNN does not detect two small TLs (denoted by FN) and mis-classifies two TLs (denoted by FP). On the contrary, the proposed method effectively recognizes small TLs. These trends can be observed in other rows in Figure 5. The result shown in the last row reveals that the proposed and conventional methods mis-classify ’green-left’ into ’green’ (denoted by FP). The percentages of ’green-left’ and ’green’ in dataset are 1.67% and 48.43%, respectively. This implicates that the ’green-left’ data need to be supplemented in the dataset. One interesting result is that the proposed method gives a higher TL confidence score than the conventional method, even when Faster R-CNN also recognizes a TL.

## 4. Conclusions

In this study, we propose a two-staged deep-learning-based traffic light recognition method that consists of candidate detection and classification stages. To efficiently reduce the number of weight parameters and computational complexity, a semantic segmentation technique and a fully convolutional network (FCN) are applied. A binary-semantic segmentation network is proposed to detect small-size traffic lights. We also propose a novel traffic light classification network including a convolution layer with three filters of (1 × 1). The simulation results show that the proposed traffic light recognition method outperforms the conventional Faster R-CNN in terms of recognition performance, and it remarkably reduces the computational complexity and hardware requirements. The traffic light recognition method achieves up to 44.5% in overall mAP and 70.16% in mAP@0.5. Especially, the empirical results show that the proposed method gives great improvement for the detection and recognition of small TLs. The proposed method can also be implemented in real-time processing with the sacrifice of a minor decrease in recognition performance. This framework can be a powerful network design guideline for the detection and recognition of small objects like traffic lights. Further research is to improve the recognition performance for “green-left” and “yellow” TLs, which occur in very short period of time.

## Figures and Tables

**Table 1:** Analysis of traffic light size in evaluation dataset.

Traffic Light’s Size	Proportion (%)	Width [pixel]			Height [pixel]		
		Min	Max	Mean	Min	Max	Mean
Small (area $<32^{2}$)	89.01	2	40	9.89	4	76	23.81
Non-small ($32^{2}\leq$ area)	10.99	15	99	27.39	24	208	62.45
Total	100.00	2	99	**11.14**	4	208	**26.56**

**Table 2:** TL candidate detection performances.

Measure Metrics	Faster R-CNN			BSSNet-Full-Size			BSSNet-Half-Size		
	Total	Small	Non-Small	Total	Small	Non-Small	Total	Small	Non-Small
No. of Traffic Lights (GT)	4306	3815	491	4306	3815	491	4306	3815	491
No. of True Positive	3685	3229	456	**4088**	**3597**	**491**	3908	3417	**491**
No. of False Negative	621	586	35	**218**	**218**	**0**	398	398	**0**
No. of False Positive	2619	1933	686	203	139	64	**128**	**99**	**29**
Precision (%)	58.45	62.55	39.93	95.27	96.28	88.47	**96.83**	**97.18**	**94.42**
Recall (%)	85.58	84.64	92.87	**94.94**	**94.29**	**100.00**	90.76	89.57	**100.00**
F-measure (%)	69.46	71.94	55.85	**95.10**	**95.27**	93.88	93.69	93.22	**97.13**

**Table 3:** TL recognition performances (overall mAP and overall AP) on test set.

TL Recognition Method	Overall mAP (%)			Overall AP (%)					
	Total	Small	Non-Small	Green	Red	Yellow	Red-Left	Green-Left	Off
Faster R-CNN [40]	20.40	15.85	36.15	33.46	23.81	4.75	34.69	17.59	8.08
BSSNet-full-size & TLC1Net	41.00	34.94	69.09	49.97	35.19	24.66	57.03	51.00	28.12
BSSNet-full-size & TLC2Net	42.96	36.93	72.31	52.07	37.79	26.47	59.04	53.00	29.39
BSSNet-full-size & TLC3Net	**44.50**	**38.98**	**75.52**	**53.91**	**39.16**	**27.82**	**61.20**	**54.36**	**30.52**
BSSNet-half-size & TLC1Net	31.04	25.55	66.53	41.81	25.5	19.34	47.38	38.76	13.46
BSSNet-half-size & TLC2Net	34.22	28.70	70.01	43.18	28.42	21.52	53.21	42.74	16.25
BSSNet-half-size & TLC3Net	36.32	30.69	73.62	44.80	29.60	22.65	54.74	48.02	18.10

**Table 4:** TL recognition performances (mAP@0.5 and AP@0.5) on test set.

TL Recognition Method	mAP@0.5 (%)			AP@0.5 (%)					
	Total	Small	Non-Small	Green	Red	Yellow	Red-Left	Green-Left	Off
Faster R-CNN [40]	38.48	31.27	57.79	70.56	52.12	8.49	59.11	27.13	13.44
BSSNet-full-size & TLC1Net	65.32	59.04	82.86	85.47	70.64	41.88	74.28	65.89	53.73
BSSNet-full-size & TLC2Net	67.67	62.26	86.65	88.05	72.92	45.71	76.95	67.48	54.93
BSSNet-full-size & TLC3Net	**70.16**	**64.88**	**89.99**	**90.66**	**74.89**	**48.77**	**79.67**	**69.75**	**57.20**
BSSNet-half-size & TLC1Net	50.67	44.94	79.60	76.58	54.43	31.59	63.29	50.36	27.78
BSSNet-half-size & TLC2Net	54.35	48.61	83.41	77.94	57.19	34.38	70.55	55.15	30.87
BSSNet-half-size & TLC3Net	57.73	52.19	87.90	80.55	59.26	36.18	72.45	62.16	35.79

**Table 5:** TL recognition performances on test dataset in LISA database.

TL Recognition Method	mAP@0.5 (%)			AP@0.5 (%)					
	Total	Small	Non-Small	Go	Go-Left	Warning	Warning-Left	Stop	Stop-Left
Faster R-CNN	44.34	40.05	48.63	53.22	34.84	42.97	33.97	52.26	43.78
BSSNet-full-size & TLC3Net	**78.31**	**65.16**	**91.47**	**83.24**	**70.19**	**83.41**	**69.86**	**88.12**	**75.07**

**Table 6:** Hardware requirements according to the TL recognition.

TL Recognition Method	Network Size [MB]	Comparison
Faster R-CNN	242.2	1x
BSSNet-x-size & TLC1Net	2.04	0.0084x
BSSNet-x-size & TLC2Net	2.05	0.0085x
BSSNet-x-size & TLC3Net	**1.97**	**0.0081x**

**Table 7:** Detail network size for each sub-network of functional stage.

Functional Stage	Sub-Network	# of Weight Parameters	Network Size [MB]
TL Candidate Detection	BSSNet-full-size	366,482	1.76
	BSSNet-half-size	366,482	1.76
TL Classification	TLC1Net	65,807	0.28
	TLC2Net	65,816	0.29
	TLC3Net	42,687	0.21

**Table 8:** Computational complexity according to TL recognition.

TL Recognition Method	Average Processing Time		Comparison
	[ms]	[fps]	
Faster R-CNN	525	1.90	1x
BSSNet-full-size & TLCxNet	96	10.42	5.47x
BSSNet-half-size & TLCxNet	**34**	**29.41**	**15.44x**
